# Effects of early ketamine exposure on cerebral gray matter volume and functional connectivity

**DOI:** 10.1038/s41598-020-72320-z

**Published:** 2020-09-23

**Authors:** Chia-Chun Hung, Yi-Hsuan Liu, Chu-Chung Huang, Cheng-Ying Chou, Chun-Ming Chen, Jeng-Ren Duann, Chiang-Shan R. Li, Tony Szu-Hsien Lee, Ching-Po Lin

**Affiliations:** 1 Institute of Brain Science, National Yang Ming University, Taipei, Taiwan; 2 Bali Psychiatric Center, Ministry of Health and Welfare, New Taipei City, Taiwan; 3 Institute of Neuroscience, National Yang Ming University, No.155, Sec.2, Li-nong Street, Taipei, Taiwan; 4 Institute of Cognitive Neuroscience, School of Psychology and Cognitive Science, East China Normal University, Shanghai, 200062 Taiwan; 5 Department of Biomechatronics Engineering, National Taiwan University, Taipei, Taiwan; 6 Department of Radiology, China Medical University Hospital, Taichung, Taiwan; 7 Institute of Education, National Chiao Tung University, Hsinchu, Taiwan; 8 Institute for Neural Computation, University of California San Diego, La Jolla, CA USA; 9 Department of Psychiatry, Yale University, New Haven, CT USA; 10 Departemnt of Neuroscience, Yale University, New Haven, CT USA; 11 Department of Health Promotion and Health Education, National Taiwan Normal University, 162 Section One, He-Ping East Road, Taipei, Taiwan; 12 CTBC Center for Addiction Prevention and Policy Research, National Taiwan Normal University, Taipei, Taiwan

**Keywords:** Neuroscience, Medical research

## Abstract

Ketamine has been used for medical purposes, most typically as an anesthetic, and recent studies support its use in the treatment of depression. However, ketamine tends to be abused by adolescents and young adults. In the current study, we examined the effects of early ketamine exposure on brain structure and function. We employed MRI to assess the effects of ketamine abuse on cerebral gray matter volume (GMV) and functional connectivity (FC) in 34 users and 19 non-users, employing covariates. Ketamine users were categorized as adolescent-onset and adult-onset based on when they were first exposed to ketamine. Imaging data were processed by published routines in SPM and AFNI. The results revealed lower GMV in the left precuneus in ketamine users, with a larger decrease in the adolescent-onset group. The results from a seed-based correlation analysis show that both ketamine groups had higher functional connectivity between left precuneus (seed) and right precuneus than the control group. Compared to controls, ketamine users showed decreased GMV in the right insula, left inferior parietal lobule, left dorsolateral prefrontal cortex/superior frontal gyrus, and left medial orbitofrontal cortex. These preliminary results characterize the effects of ketamine misuse on brain structure and function and highlight the influence of earlier exposure to ketamine on the development of the brain. The precuneus, a structure of central importance to cerebral functional organization, may be particularly vulnerable to the influences of early ketamine exposure. How these structural and functional brain changes may relate to the cognitive and affective deficits remains to be determined with a large cohort of participants.

## Introduction

Substance use disorder is a chronic brain disease with peak onset occurring during adolescence. Brain imaging studies of adolescents have suggested morphological and functional changes with early exposure to substances. For example, adolescents who abuse marijuana showed decreases in whole brain volume^1^ and more specifically in gray matter volume (GMV) in the medial orbital prefrontal cortex^2^ and bilateral hippocampus^3^. Adolescent substance abusers also showed impaired axonal connectivity compared to healthy controls^4^. Studies demonstrated changes in cortical thickness^5^, smaller cerebellar volumes^5^, decreased hippocampal volumes^6,7^, and altered white matter integrity^8^ in binge-drinking adolescents relative to non-drinkers. In a prospective study, heavy-drinking adolescents showed accelerated gray matter reduction in lateral frontal and temporal cortical GMV and attenuated white matter growth of the corpus callosum and pons relative to nondrinkers^9^. In an fMRI study, abstinent adolescent marijuana users, as compared to non-drug using controls, showed altered brain activation during working memory^10–12^, verbal learning^13^, and response inhibition^14^. Adolescents with heavy alcohol use exhibited impaired visuospatial memory^15^ and executive function^16,17^ as well as deviated brain activation during visual working memory^15,18^ and verbal encoding^19^ tasks, in contrast to controls. Together, these studies provide ample evidence for structural and functional brain changes in adolescent substance abusers.

Long used as an anesthetic agent for surgery, ketamine has recently been approved to treat patients with refractory depression. On the other hand, recreational use of ketamine is rapidly becoming a serious public health issue in East and Southeast Asia, including Taiwan. Since the 1990s^20^, ketamine has been the most common abused substance in the young population. Among toxicology consultations in the year 2010, according to the Hong Kong Poison Information Centre (HKPIC), ketamine misuse-related acute/chronic complications ranked as the 4th most common etiology^21^. In Taiwan, a survey in 2006 shows that ketamine ranked among the top three illegal substances in school-attending adolescents, together with MDMA and marijuana^22^. In the National Household Survey on health and substance abuse conducted by the Taiwanese government, ketamine ranked third (22%) and second (54%) as the most used illicit substance in people between 12 and 64 years of age in 2005 and 2009 respectively^23,24^. The average age of first ketamine use usually falls in the junior high school period^25^, a critical period for brain development and maturation. Ketamine is much cheaper than other recreational drugs and does not appear to produce immediate, serious side effects or severe withdrawal symptoms. These characteristics may have led to its widespread use, particularly among adolescents and young adults.

On the other hand, little is known about the impacts of chronic ketamine exposure on the brain. Structurally, compared with health controls, decreased GMV in bilateral frontal cortex was reported in chronic ketamine users, negatively correlated with lifetime ketamine consumption^26^. Another study revealed multiple cortical atrophies after heavy ketamine use for years, and not only in the frontal lobes^27^. This may imply broader ketamine effects on the brain. In a study with diffusion tensor imaging analysis, the ketamine users were observed to have significant reductions in fractional anisotropy over the bilateral frontal cortex and left temporoparietal cortex^28^. Edward Roberts et al. studied 17 recreational ketamine users and reported widespread reductions in axial diffusivity of the right prefrontal cortex (anterior corona radiata and forceps minor) compared with the controls. The dissociative symptoms in ketamine users were related to differences in connectivity between the caudate and the prefrontal cortex^29^. Functionally, Liao et al. reported significantly less connectivity between the thalamus and the cortical regions, including the prefrontal cortex, the motor cortex/supplementary motor area, and the posterior parietal cortex of ketamine users, compared to healthy subjects^30^. In our previous functional imaging analysis of ketamine users, compared to controls, ketamine users showed higher connectivity between the caudate and dorsal anterior cingulate cortex and between the pallidum and bilateral cerebellum. In ketamine users, connectivity of the putamen was associated with both impulsivity and duration of ketamine use^31^. In a study focused on depression, ketamine users showed less sgACC connectivity to the orbitofrontal cortex (OFC) with increasing depression severity^32^. To our knowledge, no studies have investigated the influence of early ketamine exposure on cerebral structure and function. In this study, we examined structural and functional changes in the brains of chronic ketamine abusers, with a specific focus on distinguishing the effects of adolescent vs adult onset of ketamine use.

## Methods

### Participants and assessments

The Research Ethics Committee of the China Medical University Hospital approved the study protocol (CMUH103-REC2-052). This study was performed in accordance with the ethical guidelines and regulations suggested by the International Committee of Medical Journal Editors (ICMJE) such as voluntary participation, privacy protection, and assurance of confidentiality.

Ketamine user (KU) and healthy control (HC) participants were recruited through posters at hospitals and online advertisements in the greater Taichung City, Taiwan. Candidates were assured at screening that their decision to participate in the study or not would not affect their right to medical care, that all personal information would be kept confidential, and that they could withdraw from the study at any time. Each participant provided a written informed consent prior to data collection.

After consenting to the study, participants completed a clinical diagnostic interview with a psychiatrist, questionnaire assessments, and magnetic resonance imaging (MRI)^32^. The KUs met the International Statistical Classification of Diseases and Related Health Problems (ICD) criteria for ketamine use disorders and tested positive for ketamine through urine toxicology^32^. Positive test results for other substances, namely methamphetamine, opioids, ecstasy, and/or marijuana, were the exclusion criteria^32^. All HC participants denied use of any illicit substances and showed negative urine test results^21^. None of the KU or HC participants had any major medical or neurological illnesses, history of brain concussion that resulted in loss of consciousness, or other psychiatric disorders^32^. A total of 36 KUs and 20 HCs participated in the study, although 2 KUs and 1 HC were later excluded due to poor image quality, resulting in 34 KUs and 19 HCs for analysis. The 17 KUs who initiated use of ketamine before age 20 were assigned to the adolescent-onset group and the 17 who started to use ketamine after age 20 were assigned to the adult-onset group.

All participants underwent brain structural/functional MRI scans and clinical assessments based on the Barratt Impulsiveness Scale (BIS-11)^33^, the Buss-Perry Aggression Questionnaire (BPAQ)^34,35^, The 10-Item Center for Epidemiologic Studies Depression Scale (CES-D-10)^36^, and the Sensitivity to Punishment/Sensitivity to Reward Questionnaire (SPSRQ)^37,38^.

### Magnetic resonance imaging

The 6-min resting-state fMRI and high-resolution structural imaging results were acquired using a 3-T scanner (Signa HDx, GE, Milwaukee, USA) at the Department of Radiology, China Medical University Hospital, Taichung, Taiwan. A three-dimensional spoiled gradient-recalled protocol was employed, with an inversion-recovery pulse-prepared (3D-SPGR-IrP) sequence (parameters: TE = minimal; prep time = 450 ms; flip angle = 12°; image matrix = 224 × 224; FOV = 224 mm × 224 mm; slice thickness = 1 mm; NEX = 1) and a gradient-echo, single-shot-echo planar imaging sequence (parameters: TE = 35 ms; TR = 2000 ms; slice thickness = 4.4 mm; slice number = 32; image matrix = 64 × 64; FOV = 240 mm; total scan time = 10 min)^31,32^.

### Voxel-based morphometry analysis

Diffeomorphic anatomical registration through exponentiated lie algebra-based (DARTEL) T1 VBM analysis^39^ was used to process T1 images using Gaser’s VBM8 toolbox (https://dbm.neuro.uni-jena.de), a component of Statistical Parametric Mapping (SPM8; Wellcome Institute of Neurology, University College London, UK). The processing includes the following procedures: segmentation, affine registration, DARTEL template creation, modulation for the GM, WM, and CSF tissue maps, normalization to the standard MNI space (Montreal Neurological Institute), and of smoothing and segmented tissue volumes (i.e., GM and WM) were estimated. The procedures were respectively as follows: (1) the brain image was divided into segments of the GM, WM, and CSF in the native space; (2) the native space segments were affine-registered to the tissue probability maps in the Montreal Neurological Institute (MNI) standard space; (3) the DARTEL toolbox was used to create the group template from the affine-registered GM and WM tissue segments of all participants; (4) the GM, WM, and CSF tissue maps were modulated by the nonlinear deformation parameters obtained in the previous step; (5) the modulated segments were converted to an isotropic voxel resolution of 1.5 × 1.5 × 1.5 mm; (6) finally, before the voxel-wise group comparisons, all normalized, segmented, and modulated images were smoothed with an 8-mm Gaussian kernel.

### Seed-based functional connectivity analysis

The resting-state fMRI data were preprocessed using AFNI (Analysis of Functional Neuroimages, https://afni.nimh.nih.gov/), FSL v.5.0.9 (Functional MRI of the Brain Software Library, https://fsl.fmrib.ox.ac.uk/fsl/fslwiki), and SPM8 (SPM8; Wellcome Institute of Neurology, University College London, UK) software. We adopted a fMRI preprocess similar to that described in a previous paper^40^ and the human connectome project^41^. The standard preprocessing included: (1) volume removals—the first 10 volumes from the acquired rs-fMRI dataset were dropped for magnetic homogenization; (2) head movement correction (MCFLIRT^42^), the rs-fMRI data were spatially corrected by three-dimensional rigid-body motion across measurements; (3) non-brain tissue removal (BET^43^ C:\Users\wayalan\Downloads\, https:\\fsl.fmrib.ox.ac.uk\fsl\fslwiki\BET), the non-brain tissue was removed; (4) slice-timing correction (slicetimer), the temporal shifts in rs-fMRI data acquisition were corrected; (5) normalization, the preprocessed rs-fMRI data sets were registered into standard MNI space using an EPI template; (6) large transient and polynomial trend removal (3dDespike; 3dDetrend)—background noise was removed; (7) band-pass filtering (0.01–0.08 Hz) (3dbandpass)—low-frequency drifts, high-frequency noise, and nuisance signals were removed to minimize non-neural noise at the same time, with the nuisance signals being an average of the cerebrospinal fluid time series, the white matter time series, and the Friston 24-motion-parameter model^44^; (8) spatial smoothing (3dBlurToFWHMx)—the preprocessed rs-fMRI data were smoothed with a 6 mm full-width, half-maximum Gaussian kernel.

To address motion-related issues of the rs-fMRI, we ran not only the previous motion control analysis but also quantified framewise displacement (FD) to exclude participants who exhibited extreme head motion^40^. Specifically, the exclusion criteria were as follows: (1) scans with maximum head motion more than 3 mm or 3°; (2) mean FD greater than 0.4 mm; (3) need to remove more than 37.5% of the image volume after censoring of all time points with FDs > 0.4 mm. Two KUs and one HC were excluded in this latter step.

A total of 34 KUs and 19 HCs were retained for the brain image analysis after the checks for head movement. The average FD values for the healthy control subjects, adult-onset ketamine users, and adolescent-onset ketamine users were 0.10 (*SD* = 0.33), 0.12 (*SD* = 0.47) and 0.12 (*SD* = 0.39) mm respectively. There was not a significant difference among the three groups on FD, *F*(2, 50) = 1.60, *p* = 0.21.

For the FC analysis, we defined seeds based on the volume differences between groups (HC vs KU; HC vs KU adolescent onset of use vs KU adult onset of use). A 10-mm-diameter sphere centered at each coordinate represented each seed. Pearson correlations were calculated between the BOLD time series extracted from the seed and other voxels of the whole brain using the 3dROIstats. Finally, the correlation coefficient values (*r*) were converted to *z* values by using the Fisher *z* transformation.

### Statistical analysis

We mainly used SPM8 for the brain image statistical analyses and examined the different effects of age, gender, and education across groups. We controlled these items by setting them as covariates for all statistical analyses. Firstly, we analyzed brain volume and functional changes between the KU and HC groups. An analysis of covariance (ANCOVA) was performed to examine the differences in volume and FC between the HC and KU groups by adjusting for the confounding effects of age, years of education, and sex. Secondly, we further divided the KU group into two groups based on the age of first exposure to ketamine to explore the effects of different times of ketamine onset. An ANCOVA was performed to examine the differences of volume and FC between groups (HC vs. KU adolescent onset vs. KU adult onset), covarying the confounding effects of age, years of education, and sex. *F* tests were performed to examine the main effects across the three groups, and post hoc *t* tests were performed to examine the differences between pairs of groups.

For all voxel-wise statistical analyses, we addressed the problem of image-based multiple comparisons. The AlphaSim program (AFNI tool-3dClustSim, 10,000 simulations with explicit GM mask, version AFNI_18.2.18^44^) was used to determine the statistical criterion. For the VBM analyses, a double statistical threshold (*t* test: voxel-wise *p* < 0.005 and cluster size ≥ 393 voxels; *F* test: voxel-wise *p* < 0.005 and cluster size ≥ 270 voxels) was used to determine whether the *p* value met the alpha level of 0.05 corrected for multiple comparisons. For the FC analyses, a double statistical threshold (*t* test for left DLPFC: voxel-wise *p* < 0.001 and cluster size ≥ 61 voxels; *t* test for left MOFC: voxel-wise *p* < 0.005 and cluster size ≥ 125 voxels; *F* test: voxel-wise *p* < 0.001 and cluster size ≥ 61 voxels) was again used to determine whether the *p* value met the alpha level of 0.05 corrected for multiple comparisons.

Statistical analyses of participant demographic and questionnaire scores were also performed using the Statistical Package for Social Sciences (SPSS) software package (SPSS 20 for Windows, Chicago, IL, USA). Two-sample *t* tests and analysis of covariance (ANCOVA) were used to examine the differences between groups on participant demographic and questionnaire scores. Finally, partial linear correlations between clinical measures and brain morphologic/functional changes were obtained.

## Results

### Participants

Characteristics of the study samples are reported in Table 1. There are no significant differences in gender ratio and age in any of the groups except for a lower education level in the KU group. The average ketamine use duration of users was 4.82 years (*SD* = 3.14) with the first onset age at 20.70 (*SD* = 4.93). We further divided chronic ketamine users into adolescent-onset and adult-onset groups based on their first exposure to ketamine. The participants included 17 adolescent-onset KUs (mean age = 22, *SD* = 2.62 years), 17 adult-onset KUs (mean age = 29.06, *SD* = 5.9) and 19 HCs (mean age = 25.26, *SD* = 4.33). There were significant differences among the three groups in age, *F*(2, 50) = 10.56, *p* < 0.001 and years of education, *F*(2, 50) = 34.59, *p* < 0.001. Within the KU group, there were no significant differences between the two onset groups in ketamine use duration, with a mean of 5.29 years (*SD* = 2.76) for the adolescent-onset group and 4.35 years (*SD* = 0.51) for the adult-onset group, *t*(32) = 0.87, *p* = 0.39. A significant difference was seen for the onset age. The adolescent-onset group started ketamine at a significantly younger age (16.71, *SD* = 1.57) than the adult-onset group (24.7, *SD* = 3.7), *t*(32) = − 8.20, *p* < 0.001. Four questionnaires were used to assess the participants’ impulsiveness, violence, depression, and sensitivity to reward/punishment. There were no significant differences across the three groups on BIS-11, *F*(2, 50) = 1.01, *p* = 0.37; BPAQ, *F*(2, 50) = 0.03, *p* = 0.98**;** CES-D-10, *F*(2, 50) = 0.20, *p* = 0.82; and SPSRQ, *F*(2, 50) = 0.39, *p* = 0.68 (Table 2).

**Table 1:** Demographic data of participants.

Variables: mean (*SD*)	Main groups			User onset groups		
	Ketamine users (*n* = 34)	Controls (*n* = 19)	Statistics	Adolescent-onset users (*n* = 17)	Adult-onset users (*n* = 17)	Statistics
Male/female (male %)	26/8 (76)	11/8 (58)	Χ^2^ = 2.00, *p* = .16 ^a^	14/3 (82.35)	12/5 (70.59)	X^2^ = 2.55, *p* = .28 ^b^
Age, years	25.53 (5.75)	25.26 (4.33)	*t* = 0.18, *p* = .86 ^c^	22 (2.62)	29.06 (5.9)	*F* = 10.56, *p* < .001***^d^
Education, years	11.76 (1.94)	15.63 (0.68)	*t* = 10.52, *p* < .001*** ^c^	11.65 (1.69)	11.88 (2.2)	*F* = 34.59, *p* < .001***^d^
Duration of ketamine use, years	4.82 (3.14)	X	X	5.29 (2.76)	4.35 (0.51)	*t* = 0.87, *p* = .39 ^e^
Age of first ketamine use, years	20.7 (4.93)	X	X	16.71 (1.57)	24.7 (3.7)	*t* = − 8.20, *p* < .001*** ^e^

*SD* standard deviation.

***Bonferroni corrected *p* < .001.

^a^Chi-square test between ketamine users and controls.

^b^Chi-square test across adolescent-onset group, adult-onset group, and controls.

^c^Two-sample *t* test between ketamine users and controls.

^d^Analysis of variance (ANOVA) across adolescent-onset group, adult-onset group, and controls.

^e^Two-sample *t* test between ketamine users and controls.

**Table 2:** Mean self-report questionnaire scores.

Variables: mean (*SD*)	Main groups			User onset groups		
	Ketamine users (*n* = 34)	Control group (*n* = 19)	Statistics^a^	Adolescent-onset users (*n* = 17)	Adult-onset users (*n* = 17)	Statistics^b^
**Barratt impulsiveness scale (BIS-11)**						
Total score	68.5 (2.0)	63.7 (3.0)	*F* = 1.01, *p* = .37	70.2 (2.8)	66.9 (2.7)	*F* = 1.01, *p* = .37
Attentional impulsiveness	18.5 (0.6)	17.1 (0.9)	*F* = 0.99, *p* = .38	19.0 (0.9)	17.9 (0.8)	*F* = 0.99, *p* = .38
Motor impulsiveness	24.3 (0.8)	23.3 (1.2)	*F* = 0.32, *p* = .73	24.7 (1.1)	23.9 (1.1)	*F* = 0.32, *p* = .73
Non-planning impulsiveness	25.8 (0.9)	23.3 (1.4)	*F* = 1.02, *p* = .37	26.5 (1.3)	25.1 (1.3)	*F* = 1.02, *p* = .37
**Buss–Perry aggression questionnaire (BPAQ)**						
Total score	67.8 (3.0)	67.4 (4.7)	*F* = 0.03, *p* = . 98	68.5 (4.3)	67.2 (4.2)	*F* = 0.03, *p* = .98
Physical aggression	19.6 (1.2)	17.8 (1.8)	*F* = 0.31, *p* = .74	20.0 (1.7)	19.3 (1.6)	*F* = 0.31, *p* = .74
Verbal aggression	13.1 (0.7)	15.4 (1.1)	*F* = 1.19, *p* = .31	13.5 (1.0)	12.7 (1.0)	*F* = 1.19, *p* = .31
Anger	17.5 (1.0)	15.4 (1.6)	*F* = 0.45, *p* = .64	17.6 (1.4)	17.4 (1.4)	*F* = 0.45, *p* = .64
Hostility	17.6 (1.1)	18.8 (1.7)	*F* = 0.15, *p* = .86	17.4 (1.5)	17.8 (1.5)	*F* = 0.15, *p* = .86
**10-Item center for epidemiological studies depression scale (CES-D)**						
Total score	8.9 (1.1)	7.6(1.8)	*F* = 0.20, *p* = .82	8.5 (1.6)	9.3 (1.6)	*F* = 0.20, *p* = .81
**Sensitivity to punishment and sensitivity to reward questionnaire (SPSRQ)**						
Total score	70.6 (1.7)	73.8 (2.7)	*F* = 0.39, *p* = .68	71.1 (2.5)	70.2 (2.4)	*F* = 0.39, *p* = .68
Sensitivity to punishment scale	35.2 (1.2)	37.1 (1.8)	*F* = 0.49, *p* = .61	35.9 (1.6)	34.5 (1.6)	*F* = 0.49, *p* = .61
Sensitivity to reward scale	35.4 (1.0)	36.7 (1.5)	*F* = 0.21, *p* = .81	35.2 (1.4)	35.7 (1.3)	*F* = 0.21, *p* = .81

*SD* standard deviation.

^a^Analysis of covariance (ANCOVA) between ketamine users and controls after adjustment for age, sex, and education year.

^b^Analysis of covariance (ANCOVA) across adolescent-onset group, adult-onset group, and controls after adjustment for age, sex, and education year.

### KU versus HC

**Figure 1:**
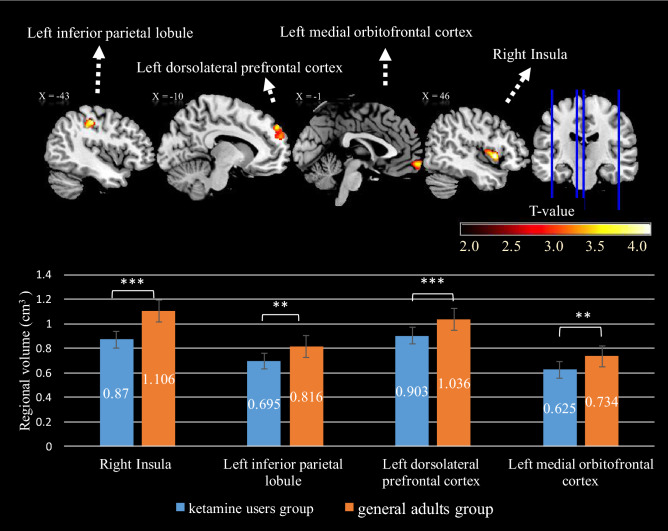
T-score map shows significant smaller gray matter volume in ketamine users than general adults. The clusters were calculated by Monte Carlo simulation with all significant criteria corrected to *p*_alpha_ < .05. Bottom bar graph shows that ketamine users have decreased effect in the right insula, left inferior parietal lobule, left dorsolateral prefrontal cortex, and left medial orbitofrontal cortex. The significance level of the analyses were corrected with age, gender, and years of education as covariates. ***LSD-corrected *p* < .001. **LSD-corrected *p* < 0.01. (Both post hoc tests follow analysis of covariance).

As shown in Table 3 and Fig. 1, independent *t* tests revealed a significantly lower (*p* < 0.001) dosage effect on the volume of the right insula in the KU group (0.87, *SD* = 0.03) than in the HC group (1.11, *SD* = 0.05); a significantly lower dosage effect (*p* = 0.001) for the left inferior parietal lobule in the KU group (0.70, *SD* = 0.02) than in the HC group (0.82, *SD* = 0.03); a significantly lower (*p* < 0.001) dosage effect for the left dorsolateral prefrontal cortex in the KU group (0.90, *SD* = 0.02) than in the HC group (1.04, *SD* = 0.02); and a significantly lower (*p* < 0.001) dosage effect for the left medial orbitofrontal cortex in the KU group (0.63, *SD* = 0.02) than in the HC group (0.73 , *SD* = 0.02).

**Table 3:** Brain structural and functional differences between Ketamine users group (KU) and health control group (HC).

MNI Atlas coordinates			Voxel size	Gray matter anatomical region	Nearest brodmann area	Regional GM volume means in cm^3^ (*SD*)		*z*
X	Y	Z				HC	KU	
**Regional gray matter volume differences**^**a**^								
45	10	− 0	469	Right insula	BA 48	1.03 (0.13)	0.91 (0.17)	3.89
− 44	− 25	45	565	Left inferior parietal lobule	BA 3	0.77 (0.08)	0.72 (0.09)	3.60
− 12	54	39	472	Left dorsolateral prefrontal cortex	BA 9	0.99 (0.06)	0.93 (0.08)	3.46
0	64	− 11	403	Left medial orbitofrontal cortex	BA 11	0.71 (0.08)	0.64 (0.07)	3.36
**Differences in functional connectivity of left DLPFC (dorsal lateral prefrontal cortex)**^**b**^								
44	10	16	108	Right inferior frontal gyrus	BA 48	X		4.61
68	− 38	20	69	Right superior temporal gyrus	BA 22	X		3.98
**Differences in functional connectivity of left MOFC (medial orbitofrontal cortex)**^**c**^								
44	22	− 2	125	Right insula	BA 47	X		3.91
48	− 28	− 20	229	Right inferior temporal gyrus	BA 20	X		3.84

*MNI* montreal neurological institute, *SD* standard deviation.

^a^Each regional cluster was corrected for multiple comparisons using Monte Carlo simulation with corrected *p*_alpha_ < .05 (*p* < .005, cluster size > 393).

^b^Each regional cluster was corrected for multiple comparisons using Monte Carlo simulation with corrected *p*_alpha_ < .05 (*p* < .001, cluster size > 61).

^c^Each regional cluster was corrected for multiple comparisons using Monte Carlo simulation with corrected *p*_alpha_ < .05 (*p* < .005, cluster size > 125).

**Figure 2:**
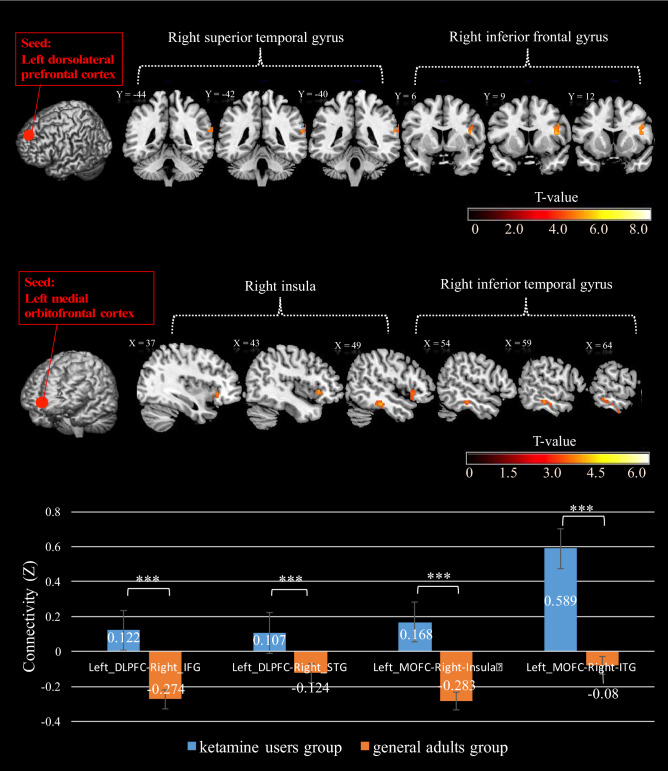
T-score map showing significant functional connectivity differences between ketamine users and healthy adults. The clusters were calculated by Monte Carlo simulation with all significant criteria corrected to *p*_alpha_ < .05. Bottom bar graph shows that ketamine users have greater effect in the left DLPFC-right IFG, left DLPFC-right STG, left MOFC-right insula, and left MOFC-right ITG. The significance level of the analyses were corrected with age, gender, and years of education as covariates. ***LSD-corrected *p* < .001. (Post hoc test follow analysis of covariance).

As for the VBM results, the left inferior parietal lobule, the left dorsolateral prefrontal cortex, and the left medial orbitofrontal cortex were defined as the seeds for the following functional connectivity analyses. As shown in Table 3 and Fig. 2, the KU group had significantly (*p* < 0.001) higher functional connectivity of the left dorsal prefrontal cortex with the right inferior frontal gyrus (0.12, *SD* = 0.04) than the HC group (− 0.27, *SD* = 0.06); and the functional connectivity with the right superior temporal gyrus was significantly higher (*p* < 0.001) in the KU group (0.11, *SD* = 0.03) than in the HC group (− 0.12, *SD* = 0.04). The results for the other seed showed a similar tendency. The KU group had significantly higher (*p* = 0.001) functional connectivity of the left medial orbitofrontal cortex with the right insula (0.17, *SD* = 0.06) than the HC group (− 0.28, *SD* = 0.09); and the functional connectivity with the right inferior temporal gyrus was significantly higher (*p* < 0.001) in the KU group (0.59, *SD* = 0.08) than in the HC group (− 0.08, *SD* = 0.11).

### Adolescent versus adult onset of use

**Figure 3:**
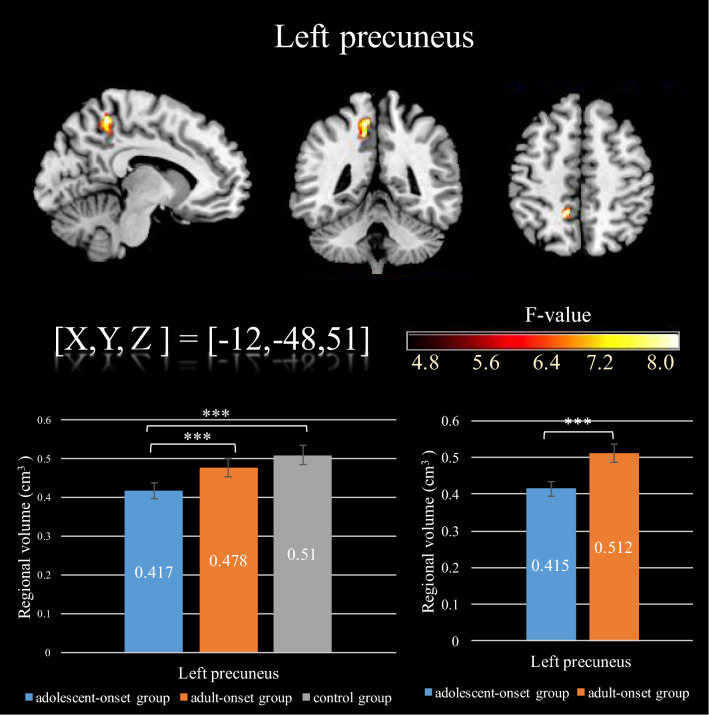
F-score map showing significant differences in left precuneus for the three groups according to ketamine use onset time. The first bar graph shows the adolescent-onset group has decreased gray matter volume in left precuneus compared to the other groups (bottom left). The differences remain after controlling for years of ketamine usage (bottom right). All of the above results were corrected for multiple comparison with all significant criteria corrected to *p*_alpha_ < .05.

These analyses revealed significant differences in gray matter volume of the left precuneus between the KU adolescent-onset group, the KU adult-onset group, and the HC group (see Fig. 3). A one-way ANCOVA was conducted to control for the effects of age, years of education, and sex, due to there being significant differences on these covariates among the three groups. There was a significant difference (*p* < 0.001) across the three groups in the volume of the left precuneus area: 0.412 (*SD* = 0.01) in the adolescent-onset group, 0.48 (*SD* = 0.01) in the adult-onset group, and 0.51 (*SD* = 0.01) in the HC group. Post hoc test results show that the left precuneus volume was significantly less (*p* < 0.001) in the adolescent-onset group than in the adult-onset group; the adult-onset group did not differ significantly (*p* = 0.13) from the control group, but the volume was significantly less (*p* < 0.001) for the adolescent-onset group than for the HC group. A further comparison was made of the two ketamine groups by controlling the number of years of ketamine use. The adolescent-onset group still had significantly (*p* < 0.001) less left precuneus volume (0.42, *SD* = 0.01) than the adult-onset group (0.51, *SD* = 0.01).

**Figure 4:**
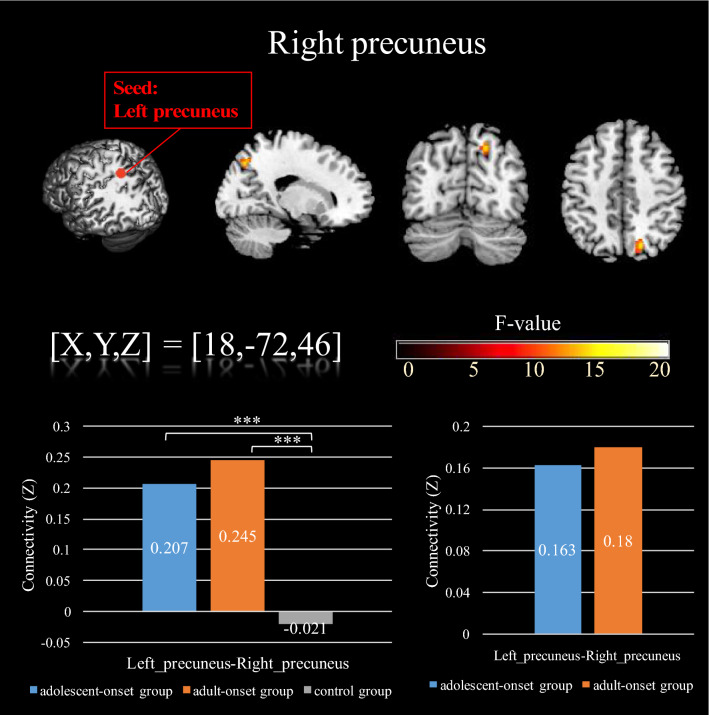
F-score map showing significant functional connectivity differences from left precuneus to right precuneus for the three groups. The first bar graph shows that the adolescent-onset group and the adult-onset group have significantly higher functional connectivity than the control group (bottom left). The group differences do not exist after controlling for years of ketamine usage (bottom right). All of the above results were corrected for multiple comparison with all significant criteria corrected to *p*_alpha_ < .05.

We used the left precuneus as a seed for a further functional connectivity analysis. Specifically, a one-way ANCOVA was conducted to control for the effects of age, years of education, and sex due to there being significant differences on these covariates across the three groups. As shown in Table 4 and Fig. 4, there was a significant difference (*p* < 0.001) across the three groups in the functional connectivity between the left precuneus area and right precuneus area: 0.21 (*SD* = 0.03) in the adolescent-onset group; 0.25 (*SD* = 0.03) in the adult-onset group; and -0.02 (*SD* = 0.03) in control group. The SCA results show that both ketamine use groups had higher functional connectivity between the left precuneus (seed) and right precuneus than the HC group. Post hoc test results show that the adolescent-onset group was significantly higher than the HC group (*p* < 0.001) and the adult-onset group was significantly higher than the control group (*p* < 0.001). There was no significant difference between the two onset groups (*p* = 0.34).

**Table 4:** Regional gray matter volume differences among the adolescent-onset group, adult-onset group, and control group.

MNI atlas coordinates			Voxel size	Gray Matter anatomical region	Nearest brodmann area	Regional GM volume mean in cm^3^ (*SD*)			*F*
X	Y	Z				Adolescent-onset group	Adult-onset group	Control group	
**Regional gray matter volume differences**^**a**^									
− 12	− 48	51	291	Left precuneus	BA 5	0.45 (0.05)	0.48 (0.05)	0.48 (0.05)	9.54
**Differences in functional connectivity between left and right precuneus**^**b**^									
18	− 72	46	65	Right precuneus	BA 7	X			13.08

*MNI* montreal neurological institute, *SD* standard deviation.

^a^Each regional cluster was corrected for multiple comparisons using Monte Carlo simulation with corrected *p*_alpha_ < .05 (*p* < .005, cluster size > 270).

^b^Each regional cluster was corrected for multiple comparisons using Monte Carlo simulation with corrected *p*_alpha_ < .05 (*p* < .001, cluster size > 61).

## Discussion

Our results demonstrate that chronic ketamine users, compared with healthy controls, had decreased brain volume over the right insula, left DLPFC, left OFC, and left inferior parietal cortex, with increased functional connectivity between the left DLPFC and right inferior frontal/superior temporal gyrus, and the left OFC and right insula/inferior temporal gyrus. The adolescent-onset group had significantly smaller left precuneus volume than the adult-onset group. Both the adolescent-onset and adult-onset groups had increased functional connectivity between the left and right precuneus.

Despite the ketamine abuse problem prevalent in some countries, few previous studies have examined changes in regional brain volume changes in chronic ketamine users. Wang et al. used structural MRI to examine 21 chronic ketamine users and showed evident cortical atrophy over the frontal, parietal, or occipital cortices^27^. Liao et al. recruited 41 ketamine-dependent subjects and reported decreased left superior frontal gyrus and right middle frontal gyrus volume compared to healthy controls in a VBM study^26^. In the present study, we demonstrated decreased brain volume over the right insula, left DLPFC, left OFC, and left inferior parietal cortex in chronic ketamine users. Decreased volume of dorsal prefrontal cortex in chronic ketamine users has been reported repeatedly in brain morphology studies, a finding consistent with observations from animal studies of a neurotoxic ketamine effect in the prefrontal cortex^45,46^.

Moreover, our data show increased functional connectivity between the left DLPFC and right inferior frontal/superior temporal gyrus, and the left OFC and right insula/inferior temporal gyrus, in chronic ketamine users compared to healthy controls. There has been little research on resting-state functional MRI in chronic ketamine users. Liao et al. focused on the thalamus of chronic ketamine users and reported significantly less resting state connectivity of the thalamus with the prefrontal cortex, motor/supplementary motor area, and posterior parietal cortex as compared to healthy controls^30^. Interestingly, in resting fMRI and PET studies, acute ketamine infusion on health subjects also showed perturbed frontal activity^47–49^. Besides, a PET study provided evidence that dorsolateral prefrontal cortex D1 receptor availability was significantly up-regulated in chronic ketamine users relative to comparison subjects^50^. Imaging studies of the impact of acute ketamine infusion on frontal responses to cognitive challenges have repeatedly shown perturbations. These have been observed across a range of domains, including working memory^51,52^, verbal fluency^53^, and memory encoding and retrieval^53,54^, as well as associative learning^55^. Together, the results of these studies provide evidence that the frontal lobe may be one of the most vulnerable brain regions to both acute and chronic ketamine exposure.

Although the information is not yet abundant, behavioral measurements in studies of cognitive function in chronic ketamine users show impaired verbal learning, verbal fluency, cognitive processing speed^56^ and spatial working memory^57^, implying disturbance of frontal functions as well. In our study, we focused on behavioral measurements of impulsivity and depression, which typically characterize patients with addiction disorders. Though the current study did not demonstrate a link between frontal gray matter attenuation and behavioral measurements (BIS-11, BPAQ, CES-D-10, SPSRQ), increased functional connectivity over DLPFC and OFC may imply a compensatory process of brain function in the chronic ketamine users in our study. Studies specific to cognitive function of the prefrontal lobe, such as emotional regulation, might provide more important evidence of chronic ketamine effects. Besides, chronic ketamine abusers may not have the impaired impulse control that has been assumed. Further comprehensive evaluation of clinical profiles of chronic ketamine users would be helpful for piloting research directions.

On the other hand, we demonstrated an onset-age effect of chronic ketamine use through significantly smaller left precuneus volume in the adolescent-onset group. Adolescence is a period of brain maturation characterized by greater plasticity and vulnerability. Previous studies that examined the vulnerability of the developing brain to neurotoxic consequences found that, compared to later onset groups, adolescents with early exposure to marijuana had poor performance on measures of verbal IQ^58^, attention^59^, impulse control^8,59^, and executive functions^59^. Moreover, studies have shown that subjects who initiated substance abuse during adolescence had more severe dependence^60^, poorer cognitive performance^59,61–63^, more profound brain morphological and functional changes^64,65^, more comorbidities^66^, and worse prognoses^67,68^ than those who began their use of drugs in adults. Our results suggest that early ketamine exposure has more impact on the developing brain, a result consistent with previous findings. Another study suggests that pre-existing neural features may increase substance use during adolescence and that drug/alcohol use during adolescence is associated with deviated neural development and impaired cognitive functioning^69^. We could not determine the direction of causality through our cross-sectional study; whether these neural changes precipitate early-onset substance use disorder or vice-versa is a question that requires larger longitudinal studies with precise evaluation and follow-up.

Ketamine is a non-competitive NMDA receptor antagonist. Previous studies have shown that the NMDA system plays an important role during this critical brain maturation period and involves several important cognitive functions. For example, studies have demonstrated that GABA-dependent plasticity in the PFC does not emerge until late adolescence^70,71^. Thomases et al. found that functional maturation of the ventral hippocampus in response to PFC connectivity is activity-dependent and requires sustained NMDAR-mediated glutamatergic transmission throughout adolescence^72^. Further, they carried out an animal study to demonstrate that the transient developmental disruptions during early adolescence by MK-801, an NMDA antagonist, can permanently alter the balance of ventral hippocampal and BLA regulation in PFC plasticity and diminish the functional capacity of prefrontal output^73^.

Several mechanisms are thought to be responsible for the neural effects of ketamine in the developing brain (see review^74^). First, ketamine produced greater and longer lasting blockage of NMDA receptors in immature compared to mature neurons^75^. Second, prolonged ketamine exposure produced compensatory up-regulation of NMDA receptors. This compensation may put neurons at risk of becoming more vulnerable to the excitotoxic effects of endogenous glutamate after ketamine withdrawal. Third, glutamate is crucial to certain stages of brain neuron development. Blockage of NMDA receptors in developmental stages, just for hours, could trigger widespread programmed cell death^76,77^. Fourth, ketamine and its metabolites could result in severe cystitis through unknown complicated immunomodulation/inflammation processes^78^; it may induce toxic effects on brain neurons as well. These effects are paths to greater vulnerability to ketamine-induced neurotoxicity in neurons of the developing brain. However, nearly all these data came from animal studies. Although these data provide clues about how the developing brain cells respond to ketamine influence, how the immature brain responds to prolonged or repeated ketamine exposure still needs further exploration.

In this study, we demonstrated significantly smaller left precuneus volume in adolescent-onset ketamine users than in adult-onset ketamine users and healthy control subjects. Increased functional connectivity of the left precuneus was found in both the adolescent-onset and adult-onset groups. The precuneus comprises a central region of the default mode network^79^, which has the highest metabolic response during rest^80^ and strong connections with adjacent and remote regions^81^. A dysfunctional default mode network has been reported in chronic cocaine users^82,83^, people with Internet use disorders^84–86^, and heroin dependent patients^87^. A recent review of addiction and precuneus function summarized evidence that the precuneus has been repeatedly implicated in exteroceptive processes, which play a critical role in the conditioned cue response in addiction^88^. In addiction studies, interoception and exteroception are frequently discussed in relation to repeated drug use behaviors. Many authors attribute sensory awareness and drug use to interoceptive processes. For example, the insular model of addiction proposed by Naqvi and Bechara^89^ is focused on the addiction process transitioning from body states to conscious feelings and to decision-making processes that involve uncertain risk and reward (e.g., practicing or avoiding physiological withdrawal). Moeller et al. illustrated the relationship between self-awareness and addictive behaviors, which also implies the role of interoception in addiction^90^. On the other hand, exteroception implies sensory awareness of outside stimuli; this awareness can also contribute to drug cravings such as, for example, positive and negative reinforcement of drug-seeking behaviors, cue-elicited cravings, and conditioned drug cues. Previous authors have considered the precuneus to be the core region of exteroceptive processes in addiction^88,91,92^, and it has been widely reported that it increases activation and connectivity in addicted populations^89^. In our study, we found decreased precuneus volume in adolescent-onset ketamine users and increased functional connectivity in chronic ketamine users regardless of use onset age. We may conclude that exteroceptive processes play a vital role in repetitive ketamine use behavior. In the clinical context, there are no severe symptoms of physical dependence from chronic ketamine use compared to use of other psychoactive drugs. Ketamine use is frequently influenced by peers. In some countries, ketamine abuse is almost exclusively limited to clubs and large parties^93^. Users report mood-elevation, relaxation, and near-death experiences as acute ketamine effects; these reactions may contribute to both positive and negative reinforcement of ketamine use.

Our results describe the effects of chronic ketamine exposure on brain structure and function and may reflect the influence of early exposure to ketamine on the development of the brain. The precuneus, a structure of central importance to cerebral functional organization, may be a key region for producing these effects. How this brain morphology and functional changes relate to the behavioral measures we employed in our study remains to be determined in future research with large cohorts of participants.
